# A Green and Innovative Waste Valorization Approach for Extraction of Flavonoids from Grapefruit Peels by Microwave-Assisted Pressurized CO_2_-H_2_O Extraction

**DOI:** 10.3390/plants14223410

**Published:** 2025-11-07

**Authors:** Hatice Neval Özbek, Hikmet Sabri Armağan, Mustafa Zafer Özel, Derya Koçak Yanık, Fahrettin Göğüş

**Affiliations:** 1 Department of Food Engineering, Engineering Faculty, University of Gaziantep, Gaziantep 27310, Türkiye; hikmetsabriarmagan@gmail.com; 2 Department of Clinical, Pharmaceutical and Biological Sciences, School of Life and Medical Sciences, University of Hertfordshire, Hatfield AL10 9AB, UK; m.ozel@herts.ac.uk; 3 Department of Food Engineering, Faculty of Agriculture, Eskişehir Osmangazi University, Eskişehir 26160, Türkiye; derya.kocakyanik@ogu.edu.tr

**Keywords:** flavonoids, microwave, pressurized CO_2_, extraction, waste

## Abstract

Grapefruit is rich in flavanones, particularly naringin and narirutin. This study investigated the effects of temperature, time, and solid-to-liquid ratio on microwave-assisted pressurized CO_2_–H_2_O (MWP-CO_2_-H_2_O) extraction of flavonoids from grapefruit and optimized the parameters for maximum total flavonoid content (TFC) using response surface methodology. Independent variable ranges were 110–160 °C, 4.00–14.00 min, and 1:10.00–1:40.00 g/mL. Optimum conditions were 128 °C, 13.88 min, and 1:31.35 g/mL, yielding a TFC of 27.96 ± 1.29 mg naringin equivalent/g dry weight. Under these conditions, extraction yield, total phenolic content, ferric reducing ability of plasma, cupric reducing antioxidant capacity, and DPPH IC_50_ were 55.17 ± 1.90% (dry basis), 25.42 ± 1.39 mg gallic acid equivalent/g, 39.16 ± 1.61 µmol trolox equivalent/g, 81.64 ± 0.29 µmol trolox equivalent/g, and 1.60 ± 0.01 mg/mL, respectively. Compared to conventional extraction (CE), MWP-CO_2_-H_2_O produced higher TFC, phenolic content, and antioxidant activity, while reducing extraction time by 13.68-fold. These results highlight grapefruit peel waste as a sustainable source of bioactive compounds and demonstrate that MWP-CO_2_-H_2_O is an environmentally sustainable, efficient alternative to conventional methods.

## 1. Introduction

Citrus species, which belong to the Rutaceae family, represent one of the most extensively cultivated fruits around the world. Oranges, grapefruits, lemons, and mandarins are significant, being cultivated worldwide for commercial purposes [1]. Grapefruit (*Citrus paradisi* Macf.) is mostly grown in tropical and subtropical climates [2]. According to the Foreign Agricultural Services of USDA, 6.81 million metric tons of grapefruit were produced in 2022/2023. China, Mexico, South Africa, the United States, and Türkiye are the main grapefruit producers in the world [3]. Grapefruit is mostly utilized in the food industry to produce juice and other citrus-based beverages [4]. When grapefruits are processed, half of the fruit is extracted as juice, whilst the remaining portion (45–50%) is peel waste made up of segment membranes, peels, and seeds [5]. These wastes convey an environmental danger and must be managed properly before disposal due to the numerous bioactive compounds they contain [6]. Citrus waste has special characteristics and is produced in high quantities, but management is limited due to environmental and economic concerns. Nowadays, traditional disposal methods (landfilling or incineration) for citrus peel wastes are inadequate and troublesome in terms of energy efficiency and environmental aspects [7]. Groundwater contamination and destruction of the aquatic ecosystem happen as a result of its high organic matter content, which contains substances with high chemical and biological oxygen demands, such as free sugars, polyphenols, and essential oils [7,8]. Furthermore, citrus peel wastes cause soil contamination due to their acidity, and the antimicrobial properties of their polyphenolic compounds inhibit the biodegradation of organic materials [8]. The first stage of recovery of value-added polyphenolics from citrus peel wastes is extraction. Increasing energy costs and the desire to decrease CO_2_ emissions, among other environmental issues, have drawn special attention to the need for the development of effective extraction methods for various industries [9].

Grapefruit peel is an abundant source of phenolic compounds, for example, phenolic acids and flavanones, particularly the distinctive flavanone glycosides, which are naringin, hesperidin, narirutin, and neohesperidin [10]. According to Shilpa et al. [11], the pith of grapefruit has a greater naringin concentration than the peel (including membrane), the seeds, and the juice. The sour and bitter flavor of naringin glycoside is attributed to the sugar neohesperidose (2-O-α-L-rhamnosyl-*ß*-D-glucose), which is abundant in grapefruit [12]. Naringin has demonstrated the ability to enhance the action of insulin and the absorption of glucose by cells. A significant factor in type 2 diabetes management is the decrease of insulin resistance, which can improve blood sugar regulation and control of overall glycemic levels [11]. Naringin shows strong antioxidant activity [13]. Also, it has significant therapeutic effects, which are protection against liver damage, improvement in liver enzyme levels, decrease of inflammation and oxidative stress, suppression of tumor development, decrease in cholesterol levels, and prevention of LDL oxidation [11].

Microwave-assisted extraction (MAE) represents a progressive solvent-based extraction technique that has been utilized in the extraction of flavonoids [14]. Microwave radiation is made up of electromagnetic waves, thus of an electric field and a magnetic field perpendicular to each other. Microwave energy is a non-ionizing kind of radiation that brings about molecular vibration through ion migration and dipole rotation without changing molecular structure [15]. In the case of MAE, biomolecules are extracted efficiently due to the pressure build-up inside of the biological tissues, which ruptures the cellular matrix and increases the flow of the analyte and solvent [14]. The high operating pressure and temperature of MAE enable a quick and effective extraction process. The rise in pressure and temperature speeds up the MAE as a result of the extraction solvent’s capacity to absorb microwave energy [16]. Water and CO_2_ are environmentally safe, non-flammable, non-toxic, and sustainable solvents [17]. Dissolving CO_2_ in water produces carbonic acid, which increases the acidity of the water [18]. The combination of CO_2_ and water may enhance extraction efficiency. In addition to that, the extraction solvent should be taken into account when developing a green approach and methods that yield good results with water or aqueous solutions [19].

Although microwave-assisted extraction and pressurized CO_2_ have been individually investigated in various studies, to the best of our knowledge, their combined application for flavonoid extraction has not yet been reported. Also, most previous studies in the literature have employed non-standardized conditions, making cross-comparison of extraction performance and environmental impact difficult. In this study, a standardized comparative framework was developed to evaluate both the microwave-assisted pressurized CO_2_–H_2_O (MWP-CO_2_-H_2_O) and conventional extraction (CE) methods under controlled conditions using the same raw material and analytical procedures. Therefore, the objectives of this study are (1) to examine the effects of temperature, time, and solid-to-liquid ratio on MWP-CO_2_-H_2_O extraction of flavonoids from grapefruit peel and to optimize extraction parameters for maximum flavonoid content using response surface methodology (RSM) and (2) to compare the yield, total phenolic content (TPC), and antioxidant activity of flavonoids extracted by MWP-CO_2_-H_2_O with those obtained via the CE method.

## 2. Materials and Methods

### 2.1. Chemicals

Naringin, gallic acid, Trolox^®^, 2,2-Diphenyl-1-picrylhydrazyl radical, Folin-Ciocalteu’s phenol reagent, sodium hydroxide, 2,4,6-Tris (2-pyridyl)-s-triazine (TPTZ) (≥99.0%), iron (III) chloride, sodium carbonate, 2,9-neocuproine, iron (II) chloride, ammonium acetate, methanol (≥99.9%), and ethanol (≥99.9%) were procured from Sigma-Aldrich (St. Louis, MO, USA). Diethylene glycol (≥99.5%) was purchased from Tekkim Chem. Co. (İstanbul, Türkiye). Naringin standard (purity of 90.5%) was purchased from Dr. Ehrenstorfer (Augsburg, Germany). Narirutin standard (purity of 99.9%) was purchased from MedChemExpress (Monmouth Junction, NJ, USA). Chlorogenic acid standard was purchased from TRC (Ottawa, ON, Canada). Caffeic acid standard was purchased from HPC Standards (Borsdorf, Germany). Carbon dioxide (99.99% purity) was purchased from Koçerler Ltd. Sti. (Gaziantep, Türkiye). Analytical- or chromatographic-grade reagents and solvents were all used.

### 2.2. Raw Material and Sample Preparation

Grapefruits of the type “Ray Ruby” (*Citrus paradisi* Macf.) were harvested from a citrus orchard in Adana, Türkiye, in January 2023. The seeds, segments, and pulp section of the grapefruits were removed. Grapefruit peels (including the flavedo and albedo section) were ground using a grater machine (Prostar 1000, Model AR-1044, Arzum, Istanbul, Türkiye). The grated peels were kept at −70 °C in polypropylene bags till the extraction process. The initial moisture content of grapefruit peel was determined as 81.62 ± 0.07% on a wet basis.

### 2.3. Experimental Section

#### 2.3.1. Conventional Extraction (CE)

Solid-liquid extraction, as a CE, was applied as stated by Garcia-Castello et al. [4] with modifications. Briefly, fresh grapefruit peel samples were extracted using solvent (30% ethanol in water). The temperature, extraction duration, and solid-to-liquid ratio were set at 70 °C, 190 min, and 1:10 g/mL, respectively. Following the extraction procedure, the extract was filtered through filter paper and kept at −20 °C until it could be examined further. The following formula was used to get the extraction yield (% db):

(1) $$Extraction\;yield\;\left(\%\;db\right)=\frac{dry\;weight\;of\;the\;extract}{dry\;weight\;of\;grapefruit\;peel}\times 100$$

#### 2.3.2. Microwave-Assisted Pressurized CO_2_-H_2_O Extraction

**Figure 1:**
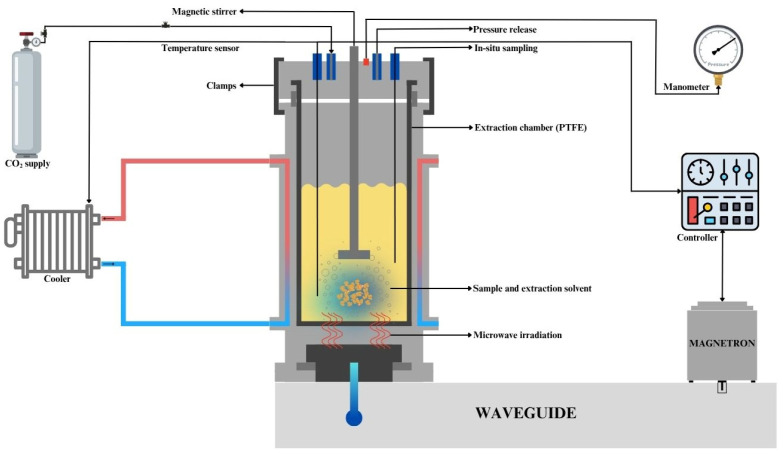
A diagram representing the microwave-assisted pressurized CO_2_-H_2_O extraction system.

Flavonoid extraction from grapefruit peel was executed in a closed vessel MWP-CO_2_-H_2_O extraction system (Milestone SynthWave, Bergamo, Italy). This system mainly consisted of an extraction vessel, gas input and exhaust, and a cooling unit (Figure 1). Grapefruit peel and cold distilled water (4 ± 1 °C) were added to the polytetrafluoroethylene (PTFE) extraction vessel, which was covered by a stainless-steel reaction chamber serving as a reaction vessel and microwave cavity. CO_2_ was utilized to pressurize the extraction chamber after it was closed. Cold distilled water was used to reach the maximum solubilization of CO_2_. A magnetic stirrer was employed, with 35% efficiency, to assist in creating an effective extraction environment during the process. The maximum power of the system is 1500 W. During the ramping phase, the power was set at 1500 W to rapidly reach the target temperature. Once the set temperature was achieved, the microwave system automatically adjusted the power between 0 and 1500 W to maintain the desired temperature throughout the extraction process. At the beginning of each run, the CO_2_ pressure was adjusted to 30.0 bar. Throughout the extraction process, the volume was kept constant at 150 mL. The MWP-CO_2_-H_2_O extraction was performed at different temperatures (110–160 °C), solid-to-liquid ratios (1:10–1:40 g/mL), and extraction times (4–14 min). These limits were chosen in order to minimize heat deterioration and guarantee effective flavonoid extraction based on preliminary experiments.

#### 2.3.3. Total Flavonoid Content

The total flavonoid content (TFC) of the extracts was analyzed using the method Huang et al. [20] outlined. Initially, 1000 µL aliquots of diluted sample were mixed with 4000 µL of distilled water, 5000 µL of diethylene glycol (90%), and 100 µL of 4 M NaOH, respectively. After a 10 min incubation in a water bath at 40 °C, the solutions were allowed to cool at room temperature. Ultimately, absorbances of the solutions were measured at 420 nm using a spectrophotometer (Pharmacia Biotech Novaspec^®^ II, Cambridge, UK). Results were reported as mg naringin equivalents/g dry weight of grapefruit peel (mg NE/g dw), with naringin (0–250 µg/mL) serving as a benchmark.

#### 2.3.4. Total Phenolic Content

The total phenolic content (TPC) of the extracts was analyzed as defined by Singleton et al. [21]. Absorbances of the solutions were read at 760 nm using a spectrophotometer. The standard was gallic acid at different concentrations (10–100 µg/mL). The findings were displayed as milligrams of gallic acid equivalents per gram of grapefruit peel dry weight (mg GAE/g dw).

#### 2.3.5. Ferric Reducing Antioxidant Power (FRAP) Assay

The FRAP content of the samples was analyzed as defined by Benzie and Strain [22]. Initially, the FRAP reagent was mixed with 300 mM sodium acetate buffer (pH 3.6), 10 mL of 1,3,5-tri (2-pyridyl)-2,4,6-triazine (TPTZ) solution (10 mM TPTZ in 40 mM HCl), and 10 mL of 20 mM iron (III) chloride solution in a ratio of 10:1:1 (*v*/*v*/*v*), respectively. Briefly, 3000 µL of FRAP reagent was combined with 100 µL of diluted extracts, and the mixture was left in a dark environment for 8 min. A spectrophotometer was then used to test the samples’ absorbance at 593 nm. The results were reported as µM Trolox equivalent/g dw of sample (µM TE/g dw), with Trolox^®^ serving as the benchmark.

#### 2.3.6. Cupric Reducing Antioxidant Activity (CUPRAC) Assay

According to Apak et al. [23], the cupric reducing antioxidant capacity test (CUPRAC) was used to assess the extracts’ antioxidant ability. A spectrophotometer was used to evaluate the samples’ absorbances at 450 nm in relation to a reagent blank solution. The results were expressed as µmol Trolox Equivalents/g dry weight of grapefruit peel (µmol TE/g dw), using Trolox^®^ as a standard.

#### 2.3.7. 2,2-Diphenyl-1-picrylhydrazyl (DPPH) Radical Scavenging Activity

The DPPH radical scavenging activity method, as outlined by Brand-Williams et al. [24], was used to quantify the extracts’ radical scavenging activity. A spectrophotometer was used to measure the solutions’ absorbances at 515 nm in relation to pure methanol, which served as a blank. The following formula was used to estimate the samples’ DPPH scavenging activity:

(2)
D
P
P
H
 
R
a
d
i
c
a
l
 
S
c
a
v
e
n
g
i
n
g
 
A
c
t
i
v
i
t
y
 %=
 
A
c
o
n
t
r
o
l−
A
s
a
m
p
l
e
A
c
o
n
t
r
o
l×
100

The flavonoid extracts obtained from MWP-CO_2_-H_2_O extraction and CE methods were evaluated using the half maximal inhibitory concentration (IC_50_) at which 50% of the DPPH radicals were scavenged. The scavenging activity was shown as mg naringin equivalents/mL extract (mg NE/mL extract).

#### 2.3.8. Qualitative Identification of Phytochemicals

The qualitative analysis of both extracts obtained from the MWP-CO_2_-H_2_O and CE methods was performed by ultra performance liquid chromatography electrospray tandem mass spectrometry (UPLC-ESI-MS/MS) (Shimadzu LC-MS/MS 8060, Kyoto, Japan) as described by Ozdemirli and Kamiloglu [25].

#### 2.3.9. Quantitative Identification of Phytochemicals

High-performance liquid chromatography–photodiode array detector (HPLC-PDA) (Shimadzu LC-2030, Kyoto, Japan) was used for the quantitative analysis of both extracts obtained from the MWP-CO_2_-H_2_O and CE methods as described by Ozdemirli and Kamiloglu [25]. The findings were reported as milligrams per gram of grapefruit peel dry weight.

### 2.4. Statistical Analysis

SPSS Statistics software (Version 26, Chicago, IL, USA) was used to analyze the data. The Design-Expert^®^ (version 13, Stat-Ease, Inc., Minneapolis, MN, USA) software’s Box-Behnken Design was used for the experimental design of the extraction of flavonoids from grapefruit peel using MWP-CO_2_-H_2_O. After the optimization process, a one-sample t-test was performed to evaluate the reliability of the predicted and experimental values of the design. One-way ANOVA was used in the comparison of the extracts obtained from the MWP-CO_2_-H_2_O and CE methods. A *p*-value below *p* < 0.05 was considered statistically significant. Every analysis was performed three times.

## 3. Results and Discussion

### 3.1. Box-Behnken Design and Optimization

#### 3.1.1. Model Fitting

RSM and Box-Behnken Design (BBD) were used to evaluate the effects of the MWP-CO_2_-H_2_O extraction process parameters of extraction temperature (X_1_), time (X_2_), and solid-to-liquid ratio (X_3_) on TFC of the grapefruit peel. Table 1 represents the design matrices of BBD along with the predicted and experimental responses. The actual variables were analyzed using multiple regression analysis. Y_TFC_ (Equation (3)) was generated as a quadratic polynomial regression model to determine the relationship between TFC and three independent variables of the design.

(3) $$Y_{TFC}=24.45+1.34X_{1\;}+1.11X_{2}+1.39X_{3}-2.06X_{1}X_{2}-3.53X_{1}X_{3}-0.235X_{2}X_{3}-2.48X_{1}^{2\;}+1.57X_{2}^{2}-2.36X_{3}^{2}$$

The significance of each variable of the design for the fitted model was determined using ANOVA and is presented in Table S1. The results showed that the model was statistically significant (*p* < 0.05) based on the F-value of 21.45 and *p*-value of 0.0003. The corresponding model and individual coefficients are considered more significant when the magnitude of the F-value is larger [26]. The determination coefficient value (R^2^) of 0.965 indicated a significant correlation between the response and the independent variables. The coefficient of determination and the adjusted coefficient of determination were 0.965 and 0.92, respectively, demonstrating a good agreement between the theoretic and experimental values. A low coefficient of variation value of 4.04% and adequate precision value of 17.69 indicated that the designed model had good accuracy, repeatability, and an adequate discrimination. A high CV (>10%) value suggests that the mean value is not developing an appropriate response model to a satisfactory degree [27]. The *p*-value of the lack of fit test was found to be 0.4670, which was non-significant (*p* > 0.05). Those statistical results indicate that a model is well fitted to the experimental data [28].

#### 3.1.2. Effect of MWP-CO_2_-H_2_O Process Parameters on TFC

**Figure 2:**
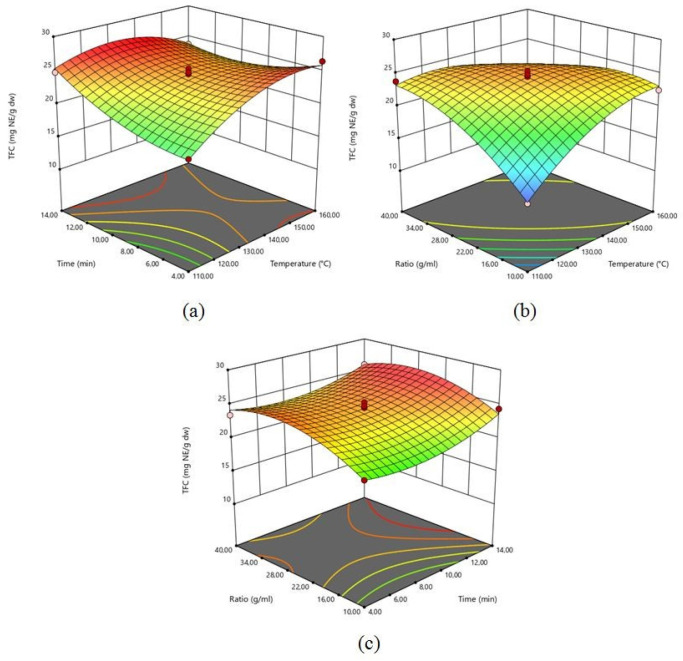
3-D response surface plots for TFC as a function of: (**a**) time and temperature; (**b**) solid-to-liquid ratio and temperature; (**c**) solid-to-liquid ratio and time.

The impact of the extraction factors (X_1_, X_2_, and X_3_) on the TFC of grapefruit peel was investigated. Table S1 shows the ANOVA results of the selected model. All process parameters (X_1_, X_2_, and X_3_) were statistically significant (*p* < 0.05) for the extraction of flavonoid from grapefruit peel. The range of TFC was 13.88 to 26.41 mg NE/g dw. The highest TFC was extracted in Run 5, with extraction temperatures of 160 °C, a solid-to-liquid ratio of 1:25.00 g/mL, and an extraction time of 4.00 min. Three-dimensional surface plots (Figure 2) explain the relationship between independent process factors and TFC. Flavonoid extraction from grapefruit peel was conducted using an extraction temperature range of 110 to 160 °C. Figure 2 clearly indicates that the increasing temperature led to increasing TFC. Increasing extraction temperature depends on microwave power being applied. By increasing microwave power, flavonoids can be extracted more easily because of microwave energy directly affecting the cell wall of the biomolecules through ionic conduction and dipole rotation. These phenomena can lead to molecular mobility, power dissipation, and heating inside the solvent and plant material [29].

In another respect, the interactive effect of extraction time and temperature (X_1_X_2_) had a statistically significant effect (*p* < 0.05) on TFC. Simultaneously increasing the extraction time and temperature had a negative effect on TFC. It can be explained due to a destructive effect. This situation was also confirmed by the coefficient of variation value (−2.06) of extraction temperature and time interaction. According to Figure 2, the quantity of extracted TFC from grapefruit peel improved with increasing extraction time. Table S1 clearly shows that the solid-to-liquid ratio has a statistically significant (*p* < 0.05) linear positive effect on TFC, which indicates that the TFC is enhanced by increasing the solid-to-liquid ratio (Figure 2). The amount of extracted TFC decreased above a solid-to-liquid ratio of 1:25.00 g/mL. Nayak et al. [30] reported similar observations for the extraction of polyphenols from orange (*Citrus sinensis*) peels using MAE. The non-uniform distribution of microwave heating [30] explains this condition. Additionally, Alara et al. [31] report similar results in MAE of flavonoids from *Vernonia amygdalina* leaf. In this research, extracted TFC increased with the decreasing feed-to-solvent ratio from 0.13 to 0.10 g/mL, but a decrease occurred in TFC when the level was below 0.10 g/mL. Large solvent volumes may require higher microwave energy absorption, which could lead to inadequate energy to break down cell walls and effectively leach out bioactive compounds [31].

#### 3.1.3. Optimization of MWP-CO_2_-H_2_O Extraction Conditions and Model Verification

To achieve the maximum TFC, the MWP-CO_2_-H_2_O extraction conditions were optimized with Design Expert software (version 13.0, Stat-Ease, Inc., Minneapolis, MN, USA). The optimum conditions of the model were found to be an extraction temperature of 128 °C, a solid-to-liquid ratio of 1:31.35 g/mL, and an extraction time of 13.88 min. Under optimum conditions, the predicted TFC was 27.51 mg NE/g dw. For the model verification, three extraction experiments were carried out at optimum conditions, and the TFC was found to be 27.96 ± 1.29 mg NE/g dw. The one-sample t-test verified that there were no statistical differences (*p* > 0.05) between the values suggested by the program and the experimental data. The results clearly show that the predicted and experimental values agreed well.

### 3.2. Comparison of MWP-CO_2_-H_2_O and CE Methods

Table S2 shows the results of a comparison between the extraction yield of MWP-CO_2_-H_2_O and CE. The extraction yield of MWP-CO_2_-H_2_O and CE was found to be 55.17% and 43.27% on a dry basis, respectively. Compared to the CE method, the MWP-CO_2_-H_2_O extraction method improved the extraction and generated a higher extraction yield. In the CE method, an ethanol and water mixture was used as a solvent, while in the MWP-CO_2_-H_2_O system, only water was employed, providing a simpler and greener extraction medium. The difference in solid-to-liquid ratios between the two methods arises from the intrinsic characteristics and optimization conditions of each process rather than a direct comparison of solvent volume. In other respects, the MWP-CO_2_-H_2_O extraction method significantly decreased (13.68-fold) the extraction time in contrast to the CE approach. The extraction times of MWP-CO_2_-H_2_O and CE methods were 13.88 min and 190 min, respectively. Similar results were found in previous studies for MAE of polyphenols (including flavonoids) from bitter orange (*Citrus aurantium*) waste [32] and flavonoid extraction from baheda (*Terminilia bellirica* Roxb.) [33]. In the MWP-CO_2_-H_2_O extraction method, the microwave irradiation causes ionic conduction and dipole rotation; these two phenomena generate molecular friction. Rapid and uniform heating can be achieved in solution due to the resistance to this electrophoretic migration of ions [34]. Raising the temperature causes the decreasing of solvent viscosity and surface tension, which increases sample wetting and matrix penetration [35]. When CO_2_ is dissolved in water, carbonic acid is formed and reduces the pH of the extraction solvent, which generates an acidic environment [17]. This can be effective in breaking down the cell wall of the plant matrix and improving the extraction of bioactive compounds. In the use of pressurized CO_2_ (30 bar) at above the boiling temperature of water, a change in the density and viscosity of the extraction solvent and CO_2_ could affect the extraction yield. Fundamentally, the MWP-CO_2_-H_2_O extraction method shows preferable process responses over the CE method.

The TFC of grapefruit peel extract found by MWP-CO_2_-H_2_O extraction and CE was compared, and Table 2 presents the findings. The TFC of the extract derived from the MWP-CO_2_-H_2_O extraction method was found to be higher (27.96 ± 1.29 mg NE/g dw) than that of the extract derived from the CE method (21.12 ± 1.07 mg NE/g dw). The TFC of the MWP-CO_2_-H_2_O extraction method was approximately 1.32-fold higher than that from the CE method, even though the MWP-CO_2_-H_2_O method has a shorter extraction time (Table S2). A statistically significant (*p* < 0.05) difference was observed in the TFC of extracts derived by MWP-CO_2_-H_2_O extraction and the CE method. This higher TFC extraction yield can be explained by considering the following phenomena; microwave heating generates a pressure inside the cells of the sample, which results in a rapid energy transfer to the solvent and plant matrix as well as an effective delivery of targeted compounds through molecular interaction with the electromagnetic field [36]. Flavonoids, particularly, are soluble in pure CO_2_, but this can be enhanced with the addition of a polar solvent (water) and by increasing the pressure of the system [37]. The combination of pressurized CO_2_-H_2_O enables the formation of carbonic acid in the extraction system. The acidic environment may enhance the migration of the flavonoid compounds from their cellular matrix, which, in turn, enhances the cleavage of phenolics bound to proteins and carbohydrate polymers [38]. Table 2 demonstrates that the TPC was lower than the TFC in both extracts. It should be noted that plant extracts with higher flavonoid content do not always have higher TPC; this could be because the Folin-Ciocalteu method alone is insufficient to measure the TFC of the targeted sample extract. Flavonoids are a subclass of phenolic compound; other phenolic compounds are classified as non-flavonoid compounds. TFC might be higher than TFC, or TPC might be lower than TFC [39]. In literature, several extraction methods for flavonoids from food and food waste have been researched. Ciğeroğlu et al. [38] studied MAE of naringin from *Citrus paradisi* Macf. Biowastes, and they reported the TFC as 13.19 mg/g dry leaf under optimum conditions. Stabrauskiene et al. [40] extracted naringin from the albedo part of grapefruit peel (*Citrus paradisi* L.) with 70% ethanol by ultrasound-assisted extraction and reported the TFC as 14.07 mg naringin/g grapefruit peel. Khan et al. [41] studied the flavonoid extraction from orange (*Citrus sinensis* L.) peel waste by ultrasound-assisted extraction and reported the TFC as 70.3 mg naringin/100 g fresh weight and 205.2 mg hesperidin/100 g fresh weight using ethanol-water mixtures. Garcia-Castello et al. [4] studied the flavonoid extraction from grapefruit (*Citrus paradisi* L.) wastes using ultrasound-assisted extraction and reported that the extracts contained 29 mg/g dw of naringin, 0.82 mg/g dw of hesperidin, 0.74 mg/g dw of narirutin, 0.17 mg/g dw of neohesperidin, and 0.017 mg/g dw of tangeritin. Additionally, the ripeness, cultivar types, and extraction process parameters could affect the flavonoid content.

TPC of grapefruit peel obtained from MWP-CO_2_-H_2_O extraction and CE methods were compared, and the results are given in Table 2. The extract derived from the MWP-CO_2_-H_2_O extraction method had significantly higher TPC (25.42 mg GAE/g dw) than the extract derived using the CE method (21.27 mg GAE/g dw) (*p* < 0.05). Typically, phenolic compounds exist in the free form or as parts of macromolecules that link with them in C-C, covalent ester, and ether bonds [42]. Phenolic compounds have an aromatic ring and contain one or more hydroxyl (-OH) groups. So, in contrast to ethanol, water is more able to dissolve phenolic compounds due to their water-soluble characteristics [39]. In the extraction space, microwaves allow for rapid heating, agitation, and H-bond breakdown by directly targeting the dipolar molecules due to dipolar rotation or ion conduction properties [43]. Additionally, microwaves, application of CO_2_, and reduced pH value of the extraction medium could enhance the cleavage of phenolic bonds (which are bound to cell walls and carbohydrates in the plant matrix), inhibit the enzyme activity to reduce the oxidation of polyphenolic compounds, and increase the extraction yield of TPC from grapefruit peel. The TPC was determined to be 19.50 mg GAE/g dw using only water as an extraction solvent by Kaanin-Boudraa et al. [44], who investigated the MAE of phenolic compounds from grapefruit peel (*Citrus* × *paradisi*). Nishad et al. [45] studied phenolic compound extraction from grapefruit peel (*Citrus paradisi* cv. Redblush) using ultrasound-assisted extraction. They found TPC as 21.16 mg GAE/g dw. In contrast, M’hiri et al. [46] stated the TPC of orange peel waste (*Citrus sinensis*) as 26.88 mg GAE/g dry orange peel.

The antioxidant activities of the extracts derived from MWP-CO_2_-H_2_O extraction and CE methods were evaluated in vitro via FRAP, CUPRAC, and DPPH-IC_50_ methods, and results are shown in Table 2. The FRAP values of the extracts for MWP-CO_2_-H_2_O extraction and CE methods were determined to be 39.16 and 25.97 µmol TE/g dw, respectively. For the MWP-CO_2_-H_2_O extraction and CE methods, the results of the CUPRAC method were found to be 81.64 and 60.07 µmol TE/g dw, respectively. The results obtained from the DPPH-IC_50_ process were 1.60 and 1.73 mg/mL for the MWP-CO_2_-H_2_O extraction and CE methods, respectively. A statistically significant (*p* < 0.05) difference was observed between MWP-CO_2_-H_2_O extraction and CE methods for antioxidant activity analysis determined by the FRAP, CUPRAC, and DPPH-IC_50_ methods. According to the results, extracts obtained from the MWP-CO_2_-H_2_O extraction method show higher antioxidant activity than the extracts obtained from the CE method. Many variables, such as the type of fruit, the extraction method applied, solvent selection, ripeness of the fruit, and the time it was harvested, might affect the antioxidant activity of plant extracts [40]. Additionally, the redox, hydrogen donating, partitioning, chelating, and radical scavenging capabilities of a compound are all factors that affect the antioxidant activity of a sample [47]. Nishad et al. [45] investigated the flavonoid and phenolic compound extraction from grapefruit peel (*Citrus paradisi* L.) and analyzed the antioxidant activity properties of extracts with FRAP (29.34 µmol TE/g dw) and CUPRAC (52.31 µmol TE/g dw) methods for CE. Park et al. [48] studied antioxidant activity properties for the edible part of blond and red grapefruit (*Citrus paradisi*) by the CUPRAC method. They reported the antioxidant activity as 30.59 and 32.62 µmol TE/g dw for blond and red grapefruit, respectively.

The relationship between the responses (TFC, TPC, FRAP, CUPRAC, and DPPH-IC_50_) of the extracts obtained from the MWP-CO_2_-H_2_O extraction method was assessed by Pearson’s correlation analysis, and the results are given in Table 3. The interaction of TFC and TPC showed a highly positive correlation coefficient value of 0.999. On the other hand, DPPH-IC_50_ did not show a positive correlation coefficient with TFC and TPC, which means that DPPH-IC_50_ decreases with increasing TFC and TPC. Decreasing DPPH-IC_50_ value shows increasing antioxidant activity in selected samples [49]. Moreover, relationships between TFC, FRAP, CUPRAC, and DPPH-IC_50_ showed strong positive correlation coefficients, which were 0.890, 0.871, and −0.959, respectively.

### 3.3. Quantitative Evaluation of Individual Phytochemicals of Grapefruit Peel Extracts Obtained from MWP-CO_2_-H_2_O and Conventional Extraction Methods

Quantitative identification of individual phytochemical compounds (flavanones and phenolic acids) obtained from MWP-CO_2_-H_2_O extraction and CE of grapefruit peel was performed and listed in Table 4. HPLC chromatograms of extracts obtained by CE and MWP-CO_2_-H_2_O extraction methods are shown in Figures S1 and S2. Naringin is the main flavanone glycoside present in the grapefruit peel extract from both methods. Naringin content was found as 25.54 and 20.51 mg/g dw for MWP-CO_2_-H_2_O extraction and CE, respectively. Results indicated that the MWP-CO_2_-H_2_O extraction method yielded higher narirutin content than the CE method, and it was found as 5.76 and 1.34 mg/g dw for MWP-CO_2_-H_2_O extraction and CE method, respectively. Goulas and Manganaris [50] studied grapefruit peel (*Citrus paradisi* Star Ruby) flavonoids and found 15.72 mg naringin/g dw and 0.84 mg narirutin/g dw. Stabrauskiene et al. [40] studied ultrasound-assisted extraction (modified with thermal hydrolysis extraction of flavonoids (naringin, narirutin, and naringenin) from the albedo and segmental part of grapefruit peel (*Citrus paradisi* L. Star Ruby) and they reported it as 14.07 mg naringin/g dw, 2.36 mg narirutin/g dw, and 0.025 mg naringenin/g dw. Unfortunately, naringenin was not found in either extract. In another research performed by Zhang et al. [51], they determined the grapefruit (*Citrus paradisi* Changshanhuyu) flavedo flavonoid composition using an HPLC-MS system, and they found it to be 3.09, 0.35, and 3.1 mg/g fresh weight for naringin, narirutin, and neohesperidin, respectively. Table 4 demonstrated that chlorogenic and caffeic acids were the main phenolic acids in both extracts obtained from MWP-CO_2_-H_2_O extraction and the CE method. Chlorogenic and caffeic acid were found as 0.30 and 0.18 mg chlorogenic acid/g dw and 0.76 and 0.31 mg caffeic acid/g dw for the CE and MWP-CO_2_-H_2_O extraction methods, respectively. Xi et al. [52] studied phenolic acids in nine varieties of grapefruit peel (*Citrus paradisi* Macf.), where chlorogenic acid ranged between15.77 and 86.34 µg/g dw, caffeic acid ranged between 0.00 and 3.88 µg/g dw, and ferulic acid ranged between 0.0 and 2.44 µg/g dw for the flavedo part of the fruit. On the other hand, the albedo part of the fruit contains little content of chlorogenic acid (0.00–5.26 µg/g dw), caffeic acid (0.0–1.68 µg/g dw), and ferulic acid (0.00–5.24 µg/g dw). He et al. [53] identified the phenolic acids, including chlorogenic and caffeic acids, from peels of the natural citrus hybrid *Citrus sinenses* L. × Citrus unshiu Marc. and *Citrus unshiu* Marc. × *Citrus clementina.* Hort ex Tanaka, and they reported 12.2 µg chlorogenic acid/g and 19.3 µg caffeic acid/g, 13.9 µg chlorogenic acid/g and 10.9 µg caffeic acid/g, and 8.8 µg chlorogenic acid/g and 11.1 µg caffeic acid/g, respectively. Differences in composition may be related to the variety of fruit sample and extraction method. According to the results, the application of the MWP-CO_2_-H_2_O process may enhance extraction of the free and bound phenolic acids from the flavedo and albedo parts of grapefruit peel. Application of microwave energy and pressurized CO_2_ can facilitate the extraction of individual flavonoids (naringin and narirutin) and phenolic acids (chlorogenic and caffeic acid) from the cellular matrix of grapefruit peel.

## 4. Conclusions

In this research, the effect of MWP-CO_2_-H_2_O process parameters of extraction temperature, solid-to-liquid ratio, and extraction time was investigated and compared with a CE for extraction of flavonoids from grapefruit peel. The findings indicated that the extraction of flavonoids from grapefruit peel was significantly impacted by independent process parameters. The solid-to-liquid ratio was the most efficacious independent process parameter on flavonoid content of the grapefruit peel extracts. The interaction of extraction temperature and time significantly impacted the TFC of the extracts. The extract obtained from MWP-CO_2_-H_2_O extraction contained a higher flavonoid content, phenolic content, and antioxidant activity than the extract obtained by the CE method. The MWP-CO_2_-H_2_O extraction method reduced extraction time by 13.68-fold over the CE method. These findings demonstrate that the combination of microwave irradiation and pressurized CO_2_ effectively enhances the recovery of flavonoids from grapefruit peel. The MWP-CO_2_-H_2_O extraction technique presents a greener and more efficient alternative to conventional methods. The term “greener” refers to the environmental advantages of this process, including the elimination of organic solvents, shorter extraction time, and lower estimated energy use. Although a formal life-cycle assessment (LCA) was not performed, future research will focus on quantitative environmental and energy evaluations to substantiate the green claims. This study also provides a standardized comparative framework that minimizes variability among different extraction techniques and supports the development of sustainable extraction systems. While the present work was conducted at laboratory scale, future studies should explore process scale-up, reactor design improvements, and techno-economic assessments to evaluate industrial applicability for citrus by-product valorization.

## Figures and Tables

**Table 1:** A Box-Behnken Design generated for MWP-CO_2_-H_2_O with predicted and experimental response values.

Run	Factors			Response	
	X_1_ (°C)	X_2_ (min)	X_3_ (g/mL)	TFC (mg NE/g dw)	
				Predicted Value	Experimental Value
1	135.00	9.00	1:25.00	24.45	24.81
2	110.00	9.00	1:40.00	23.20	23.81
3	135.00	14.00	1:40.00	25.92	25.90
4	110.00	4.00	1:25.00	19.03	19.18
5	160.00	4.00	1:25.00	25.83	26.41
6	160.00	14.00	1:25.00	23.93	23.79
7	110.00	9.00	1:10.00	13.35	13.18
8	135.00	4.00	1:40.00	23.18	23.44
9	135.00	9.00	1:25.00	24.45	24.72
10	135.00	14.00	1:10.00	23.61	23.46
11	135.00	9.00	1:25.00	24.45	25.35
12	135.00	9.00	1:25.00	24.45	24.48
13	135.00	9.00	1:25.00	24.45	22.91
14	160.00	9.00	1:10.00	23.09	22.49
15	160.00	9.00	1:40.00	18.82	18.99
16	110.00	14.00	1:25.00	25.35	24.78
17	135.00	4.00	1:10.00	20.93	20.96

X_1_: Extraction temperature; X_2_: Extraction time; X_3_: Solid-to-liquid ratio.

**Table 2:** Comparison of parameters of extracts obtained by CE and MWP-CO_2_-H_2_O extraction methods.

Outputs	Extraction Method	
	CE	MWP-CO_2_-H_2_O
TFC (mg NE/g dw)	21.12 ± 1.07 ^a^	27.96 ± 1.29 ^b^
TPC (mg GAE/g dw)	21.27 ± 0.57 ^a^	25.42 ± 1.39 ^b^
FRAP (µmol TE/g dw)	25.97 ± 0.70 ^a^	39.16 ± 1.61 ^b^
CUPRAC (µmol TE/g dw)	60.07 ± 0.48 ^a^	81.64 ± 0.29 ^b^
DPPH-IC_50_ (mg/mL)	1.73 ± 0.01 ^a^	1.60 ± 0.01 ^b^

^ab^ Significant differences exist between means with various letters within a row (*p* < 0.05).

**Table 3:** Pearson’s correlation coefficients for the MWP-CO_2_-H_2_O extraction method.

Responses	r ^a^				
	TFC	TPC	FRAP	CUPRAC	DPPH-IC_50_
TFC ^b^	1.000				
TPC ^c^	0.999	1.000			
FRAP ^d^	0.890	0.873	1.000		
CUPRAC ^e^	0.871	0.852	0.999 *	1.000	
DPPH-IC_50_^f^	−0.908	−0.892	−0.999 *	−0.997	1.000

^a^ Correlation coefficient. ^b^ Total flavonoid content (mg NE/g dw). ^c^ Total phenolic content (mg GAE/g dw). ^d^ Ferric reducing antioxidant plasma (µmol TE/g dw). ^e^ Cupric reducing antioxidant activity capacity (µmol TE/g dw). ^f^ 2,2-Dipehnyl-1-picrylhydrazyl—Half maximal inhibitory concentration (mg/mL). * Significant at *p* < 0.05.

**Table 4:** Contents of individual compounds obtained from MWP-CO_2_-H_2_O extraction and the CE method (mg/g dry weight).

Individual Compounds	Extraction Method	
	CE	MWP-CO_2_-H_2_O
Chlorogenic acid	0.18 ± 0.01 ^a^	0.30 ± 0.02 ^b^
Caffeic acid	0.31 ± 0.01 ^a^	0.76 ± 0.02 ^b^
Narirutin	1.34 ± 0.01 ^a^	5.76 ± 0.10 ^b^
Naringin	20.51 ± 0.50 ^a^	25.24 ± 0.26 ^b^
Total	22.35 ± 0.56 ^a^	32.07 ± 0.39 ^b^

^ab^ Significant differences exist between means with various letters within a row (*p* < 0.05).

## Data Availability

The data are contained within the article and the Supplementary Materials.

## Supplementary Materials

The following supporting information can be downloaded at: https://www.mdpi.com/article/10.3390/plants14223410/s1, Figure S1: HPLC chromatograms of extract obtained by CE method; Figure S2: HPLC chromatograms of extract obtained by MWP-CO_2_-H_2_O extraction method; Table S1: ANOVA results regarding the fitted quadratic polynomial model.; Table S2: Comparison of processing conditions and extraction yields of MWP-CO_2_-H_2_O and CE techniques.
