# Supplementary material for: A Green and Innovative Waste Valorization Approach for Extraction of Flavonoids from Grapefruit Peels by Microwave-Assisted Pressurized CO2-H2O Extraction
Source: Plants (Basel). 2025 Nov 7;14(22):3410. doi: 10.3390/plants14223410 (PMC12655522; doi:10.3390/plants14223410)

A green and innovative waste valorization approach for extraction of flavonoids from grapefruit peels by microwave-assisted pressurized CO_2-H_2O extraction

Hatice Neval Özbek [1,*], Hikmet Sabri Armağan [1], Mustafa Zafer Özel [2], Derya Koçak Yanık[3] and Fahrettin Göğüş [1,*]

[1] Department of Food Engineering, Engineering Faculty, University of Gaziantep, 27310, Gaziantep, Türkiye; hikmetsabriarmagan@gmail.com (H.S.A.)
[2] Department of Clinical, Pharmaceutical and Biological Sciences, School of Life and Medical Sciences, University of Hertfordshire, Hatfield, United Kingdom; m.ozel@herts.ac.uk
[3] Department of Food Engineering, Faculty of Agriculture, Eskişehir Osmangazi University, Eskişehir 26160, Türkiye; derya.kocakyanik@ogu.edu.tr
* Correspondence: haticeneval@gantep.edu.tr (H.N.Ö.); fahretgantep.edu.tr (F.G.)

Table S1

ANOVA results regarding the fitted quadratic polynomial model.

Source	TFC					
	**Sum of squares**	**df**	**Mean Square**	**F value**	***p*-value**	**Coefficient**
Model	165.88	9	18.43	21.45	0.0003[a]	
**Linear**						
X_1	14.39	1	14.39	16.75	0.0046[a]	1.34
X_2	9.77	1	9.77	11.37	0.0119[a]	1.11
X_3	15.54	1	15.54	18.09	0.0038[a]	1.39
**Interactive**						
X_1X_2	16.89	1	16.89	19.66	0.0030[a]	-2.06
X_1X_3	49.91	1	49.91	58.10	0.0001[a]	-3.53
X_2X_3	0.22	1	0.22	0.26	0.6277[b]	-0.24
**Quadratic**						
X_1^2	25.91	1	25.91	30.16	0.0009[a]	-2.48
X_2^2	10.34	1	10.34	12.03	0.0104[a]	1.57
X_3^2	23.37	1	23.37	27.20	0.0012[a]	-2.36
Lack of fit	2.63	3	0.88	1.04	0.4670[b]	
R^2	0.96					
Adj R^2	0.92					
CV	4.04					
Adeq. Prec.	17.69					

[a]significant at $p < 0.05$; [b]not significant at $p > 0.05$.

Table S2

Comparison of processing conditions and extraction yields of MWP-CO_2-H_2O and CE techniques.

**Extraction method**	**Extraction temperature (°C)**	**Extraction time (min)**	**Solid to liquid ratio (g/ml)**	**Extraction yield (% db)**
MWP-CO_2-H_2O	128	13.88	1:31	55.17 ± 1.90^{b}
CE	70	190.00	1:10	43.27 ± 1.17^{a}

^{ab}Different letters within a row indicate significantly differences ($p<0.05$).

Figure S1

HPLC chromatograms of extract obtained by CE method

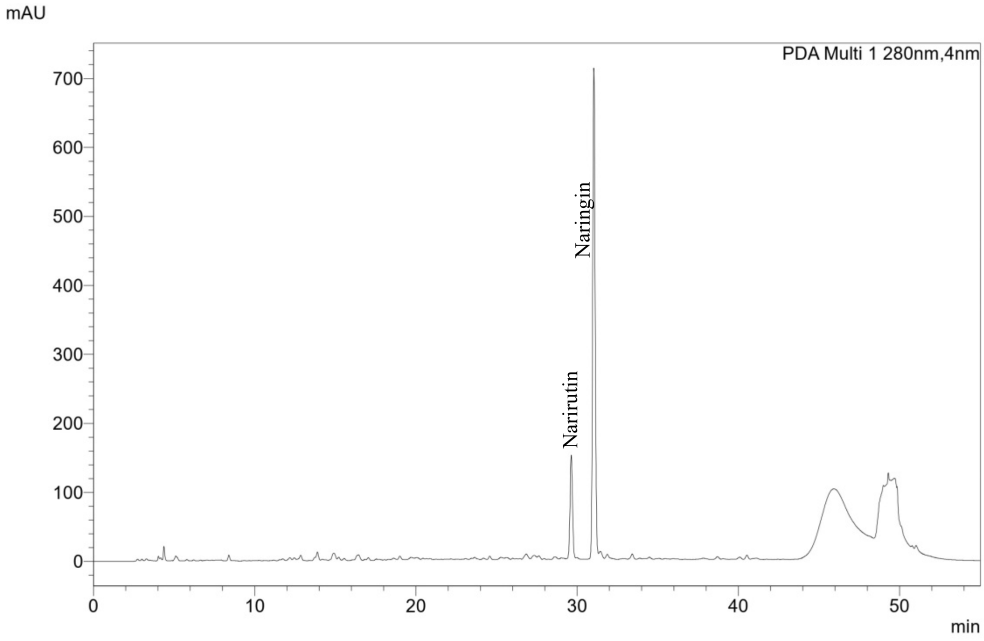

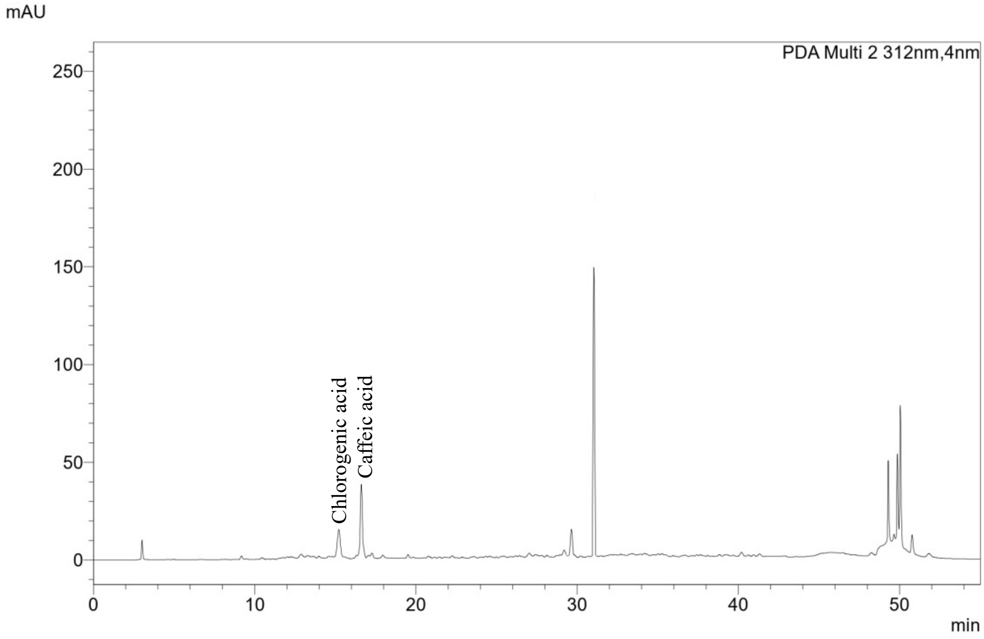

Figure S2

HPLC chromatograms of extract obtained by MWP-CO_2-H_2O extraction method

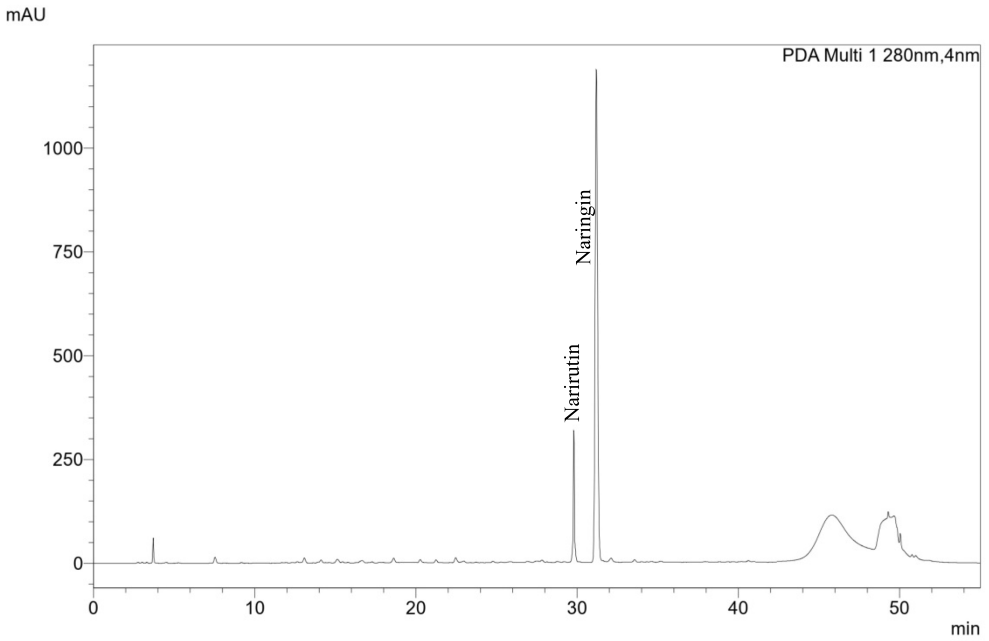

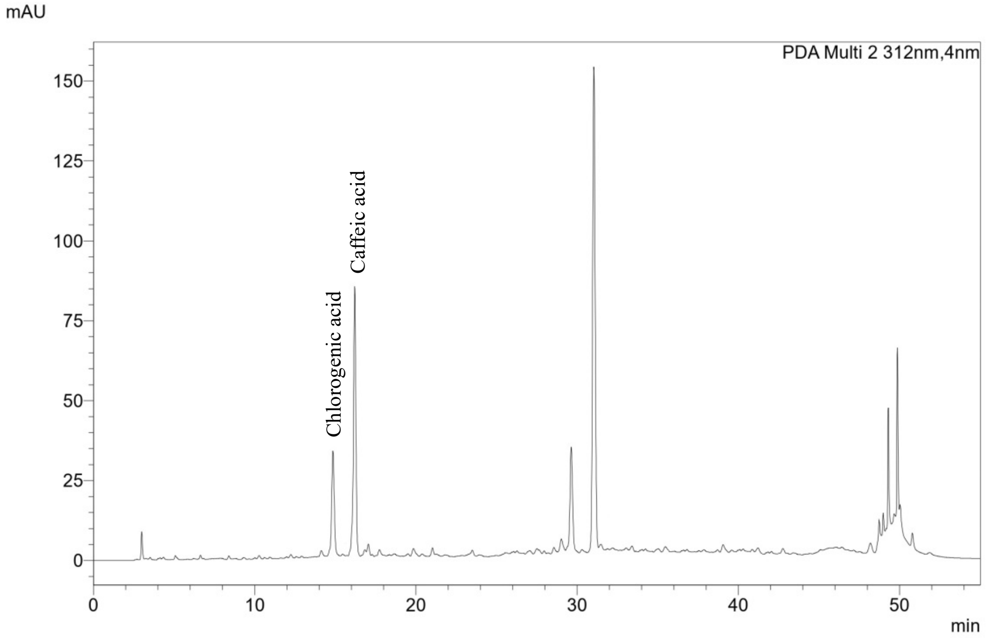

Supplement: Supplementary file 1 [file plants-14-03410-s001.zip › plants-3898743-supplementary.pdf]
